# CCDC178-mediated cytoskeleton assembly is required for spermiogenesis in mice

**DOI:** 10.1016/j.gendis.2023.101132

**Published:** 2023-09-29

**Authors:** Fucheng Dong, Xiuge Wang, Tao Huang, Yingying Yin, Sai Xiao, Yanjie Ma, Huafang Wei, Bingbing Wu, Ruidan Zhang, Liying Wang, Xuejiang Guo, Fei Gao, Chao Liu, Hongbin Liu, Jianguo Zhao, Wei Li

**Affiliations:** a State Key Laboratory of Stem Cell and Reproductive Biology, Institute of Zoology, Stem Cell and Regenerative Medicine Innovation Institute, Chinese Academy of Sciences, Beijing 100101, China; b Guangzhou Women and Children’s Medical Center, Guangzhou Medical University, Guangzhou, Guangdong 510623, China; c Shandong Technology Innovation Center for Reproductive Health, Jinan, Shandong 250012, China; d Institute of Animal Science and Veterinary Medicine, Shandong Academy of Agricultural Sciences, Jinan, Shandong 250199, China; e Key Laboratory of Reproductive Endocrinology of Ministry of Education, Shandong University, Jinan, Shandong 250012, China; f Shandong Provincial Clinical Medicine Research Center for Reproductive Health, Jinan, Shandong 250012, China; g State Key Laboratory of Reproductive Medicine Nanjing Medical University, Nanjing, Jiangsu 211166, China; h University of Chinese Academy of Sciences, Beijing 100049, China

Infertility affects around 8%–12% of couples globally, and in about 50% of these cases, male factors are either the primary cause or contribute significantly to infertility. Any defects during spermiogenesis may result in male subfertility or complete infertility in mammals.^1^ Previously, we found that CFAP53 is localized in the manchette and sperm tail, and it plays an essential role in sperm flagellum biogenesis. CFAP53 knockout leads to male infertility due to multiple morphological abnormalities of the flagella.^2^ To investigate the mechanism of CFAP53 during spermiogenesis, we searched for potential CFAP53-interacting proteins and identified a new CFAP53-interacting protein, CCDC178 (Fig. 1A). Western blotting of this protein revealed a significant decrease of CCDC178 in the testes of *Cfap53* knockout mice (Fig. S1A–C). We further showed that CCDC178 colocalized with the centrosome marker γ-tubulin and CFAP53 in either HeLa cells or HEK 293T cells (Fig. S1D). Furthermore, we found that CCDC178 is an evolutionarily conserved protein and is predominantly expressed in the testis (Fig. 1B; Fig. S1E–H), suggesting that this protein might also participate in spermiogenesis.

**Figure 1:**
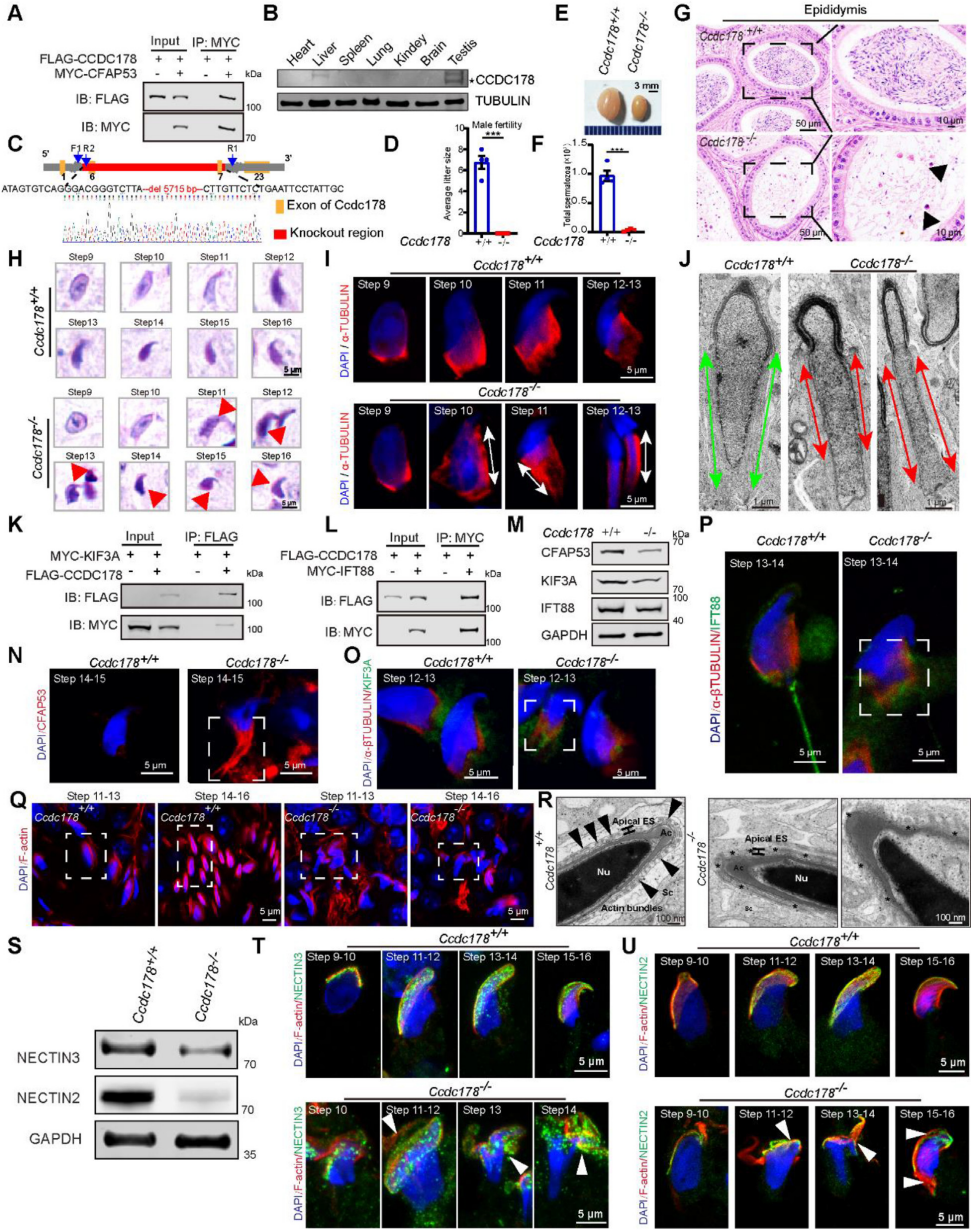
CCDC178-mediated cytoskeleton assembly is required for spermiogenesis in mice. **(A)** CCDC178 interacts with CFAP53. pRK-Flag-*Ccdc178* and pCS2-Myc-*Cfap53* were transiently co-transfected into HEK 293T cells. Cell lysates were collected for coimmunoprecipitation with anti-FLAG or anti-MYC antibody. **(B)** The expression of CCDC178 in various mouse tissues. TUBULIN was used as a loading control. **(C)** Schematic illustration of the *Ccdc178* knockout strategy. Primers F1, R1, and R2 were used for the genotyping of *Ccdc178* mice. **(D)** The average litter size of females mated with *Ccdc178*^*+/+*^ and *Ccdc178*^*−/−*^ male mice. **(E)** Representative images of testes from *Ccdc178*^*+/+*^ and *Ccdc178*^*−/−*^ mice. Each grid square represents 1 mm. **(F)** Total spermatozoa from the unilateral cauda epididymis from *Ccdc178*^*+/+*^ and *Ccdc178*^*−/−*^ mice. **(G)** Histological analyses of cauda epididymis sections from *Ccdc178*^*+/+*^ and *Ccdc178*^*−/−*^ mice stained with hematoxylin and eosin. **(H)** Morphology of spermatids at steps 9–16 in *Ccdc178*^*+/+*^ and *Ccdc178*^*−/−*^ testes stained with PAS-haematoxylin. Red arrows point to abnormal spermatids. **(I)** The manchette structure spermatids of *Ccdc178*^*+/+*^ and *Ccdc178*^*−/−*^ mice at steps 9–13. White arrows point to the abnormal elongation and destruction of the manchette in *Ccdc178*^*−/−*^ mice. Testis cells were isolated and stained with an anti-α-tubulin antibody. **(J)** Transmission electron microscopy analysis of elongated spermatids in *Ccdc178*^*+/+*^ and *Ccdc178*^*−/−*^ mouse testes. Green arrows point to manchette microtubule bundles in *Ccdc178*^*+/+*^ mice, and red arrows point to abnormal elongation and show the absence of manchette microtubules in *Ccdc178*^*−/−*^ mice. Scale bar = 1 μm. **(K, L)** CCDC178 interacts with KIF3A and IFT88. pRK-Flag-*Ccdc178*, pCS2-Myc-*Kif3a*, and pCS2-Myc-*Ift88* were co-transfected into HEK 293T cells. Cell lysates were collected for coimmunoprecipitation with an anti-FLAG antibody or an anti-MYC antibody. **(M)** Immunoblotting analysis of proteins related to the manchette in *Ccdc178*^*+/+*^ and *Ccdc178*^*−/−*^ mouse testes. GAPDH was used as the loading control. **(N)** Immunofluorescence staining of CFAP53 (red) in testis squash samples from *Ccdc178*^*+/+*^ and *Ccdc178*^*−/−*^ mice. Spermatids at steps 14–15 showed abnormal CFAP53 localization in *Ccdc178*^*−/−*^ mice (white boxes). **(O, P)** Immunofluorescence staining of KIF3A (green) and IFT88 (green) with anti-α/β-tubulin (red) on testis squash samples from *Ccdc178*^*+/+*^ and *Ccdc178*^*−/−*^ mice, respectively. Spermatids at steps 13–14 showed abnormal KIF3A and IFT88 localization in *Ccdc178*^*−/−*^ mice (white boxes). **(Q)** Analysis of the F-actin structure by phalloidin immunofluorescence (red, labeled by TRITC). Compared with the control group, spermatids at steps 11–16 showed abnormal F-actin structures in *Ccdc178*^*−/−*^ mice. **(R)** Ultrastructural analysis of the apical ectoplasmic specialization (ES) was examined by transmission electron microscopy. Arrowheads point to the actin bundles, and asterisks indicate the disrupted actin bundles. Sc, Sertoli cell; Nu, nucleus; Ac, acrosome. **(S)** The NECTIN3 and NECTIN2 protein levels in the testes of *Ccdc178*^*+/+*^ and *Ccdc178*^*−/−*^ mice were analyzed by Western blotting. GAPDH was used as a loading control. **(T)** Immunofluorescence staining with antibodies against NECTIN3 (green) and F-actin (red) in spermatids at different developmental steps from *Ccdc178*^*+/*+^ and *Ccdc178*^*−/−*^ adult mice. The nucleus was stained with DAPI (blue). **(U)** Immunofluorescence staining with antibodies against NECTIN2 (green) and F-actin (red) in spermatids of *Ccdc178*^*+/+*^ and *Ccdc178*^*−/−*^ adult mice at different developmental steps. The nucleus was stained with DAPI (blue).

To elucidate the physiological function of CCDC178, we generated *Ccdc178* knockout mice by deleting 5715 bp between exon 6 and exon 7 of *Ccdc178* (Fig. 1C). *Ccdc178* knockout resulted in complete male infertility, and their testes were smaller than those of *Ccdc178*^+/+^ mice (Fig. 1D, E; Fig. S2A–D). Furthermore, *Ccdc178*-null mice showed a dramatic reduction in sperm count (Fig. 1F, G; Fig. S2E, F), which is similar to the oligoasthenospermia patients. To determine which stages of spermiogenesis were affected by *Ccdc178* knockout, we performed PAS-haematoxylin staining of the *Ccdc178*^*−/−*^ and *Ccdc178*^+/+^ testis sections. We found that the morphology of *Ccdc178*^*−/−*^ elongated spermatid heads showed abnormalities starting from step 11 and turning to clear at step 16 during spermiogenesis (Fig. 1H; Fig. S2G, H).

We further examined the microtubule structures and observed that the manchette was present and bound to the caudal region of the elongated spermatid head, but displayed abnormal elongation and detachment from the head to the tail at steps 10–13 during spermiogenesis of *Ccdc178*^*−/−*^ mice (Fig. 1I; Fig. S3A). The manchette is known to play a crucial role in sperm head shaping and the development of the sperm flagellum.^3^ Indeed, we observed that *Ccdc178*^*−/−*^ mice are defective in the biogenesis of sperm flagella at stages VI to VIII of the seminiferous epitheliums (Fig. S3B). Transmission electron microscopy analysis further revealed aberrant elongated microtubule structures and the disruption of “9 + 2” microtubule structures (Fig. 1J; Fig. S3C). Taken together, these results indicate that *Ccdc178* knockout disrupts manchette assembly and affects sperm head shaping and flagellum biogenesis.

The proper assembly of manchette is required for intra-manchette transport and intra-flagellar transport pathways. We previously showed that CFAP53 interacts with kinesin family member 3A (KIF3A) and intra-flagellar transport 88 (IFT88) to participate in both intra-manchette transport and intra-flagellar transport during sperm flagellum biogenesis.^2^ By conducting coimmunoprecipitation experiments, we found that CCDC178 also interacted with both IFT88 and KIF3A (Fig. 1K, L). Western blotting analysis showed a significant decrease in CFAP53, KIF3A, and IFT88 in *Ccdc178*^*−/−*^ mice compared with that of *Ccdc178*^*+/+*^ mice (Fig. 1M; Fig. S3D). Furthermore, we examined the localization of CFAP53, KIF3A, and IFT88 in both *Ccdc178*^*+/+*^ and *Ccdc178*^*−/−*^ mice, and found that the expression of these proteins was affected at steps 11–13 during spermiogenesis of *Ccdc178* knockout mice, with delayed CFAP53, KIF3A, and IFT88 removal at steps 14–15 (Fig. 1N–P; Fig. S3E–G). These findings suggest that CCDC178 may participate in the assembly of manchette which is similar to that of CFAP53.

Different from *Cfap53* knockout, *Ccdc178* knockout not only leads to a phenotype like multiple morphological abnormalities of the flagella but also results in severe oligospermia in mice, suggesting that CCDC178 may also be involved in spermiation. Immunofluorescence analysis of *Ccdc178*^*−/−*^ mouse testes sections with phalloidin-TRITC labeling to visualize the actin-based cytoskeleton demonstrated a clear disruption and disorganization of the F-actin structure in spermatids at steps 11–16 (Fig. 1Q). With transmission electron microscopy, we observed that *Ccdc178*^*+/+*^ spermatids showed intact actin bundles surrounding the elongated spermatid nucleus within the apical ectoplasmic specialization structures, while *Ccdc178*^*−/−*^ spermatids displayed disrupted actin bundles, detached acrosomes, and expanded acroplaxome spaces (Fig. 1R). To investigate the potential function of CCDC178 on apical ectoplasmic specialization, we isolated the apical ectoplasmic specialization *in vitro*^4^ and examined the expression of some key apical ectoplasmic specialization adhesion proteins, NECTIN3 (expressed in elongated sperm head) and NECTIN2 (expressed in Sertoli cells and spermatids), which form NECTIN2-NECTIN3 complexes at the spermatid/Sertoli cell interface.^5^ We found that both two proteins were significantly down-regulated in *Ccdc178*^*−/−*^ mouse testes (Fig. 1S; Fig. S4C). We then conducted an immunofluorescence analysis of these two proteins in spermatids. In wild-type spermatids, both two proteins were predominantly localized on the convex side of the apical ectoplasmic specialization at steps 9–16, approaching the tip of the spermatid head, and they partially colocalized with F-actin (Fig. 1T, U; Fig. S4A, B). However, in the *Ccdc178*^*−/−*^ spermatids, the localization of either NECTIN2 or NECTIN3 was no longer restricted to the convex side of the spermatid head. Instead, they were displayed as clustered granules or dispersed distributions in spermatids at steps 11–14 (Fig. 1T, U; Fig. S4A, B). Thus, the disruption of the NECTIN3-NECTIN2 complex might contribute to the severe oligozoospermia-like phenotype in *Ccdc178* knockout mice.

In conclusion, CCDC178 is predominantly expressed in the testis and its knockout results in male infertility with an oligoasthenospermia-like phenotype. Further analysis revealed that the spermatogenic defects observed in *Ccdc178* knockout mice were caused by defects in intra-manchette transport and the disruption of the apical ectoplasmic specialization. These findings indicate that CCDC178 plays a critical role in regulating sperm head shaping and flagellum biogenesis during spermiogenesis (Fig. S4D). The conserved human CCDC178 protein may have a similar function in spermiogenesis. Although no human CCDC178 mutations have been linked to male infertility yet, our studies strongly suggest a potential association of CCDC178 mutations with the pathogenesis of oligoasthenospermia or multiple morphological abnormalities of the flagella in some patients.

## Author contributions

H.L., J.Z., and W.L. designed the experiments and revised the manuscript. F.D., X.W., and T.H. performed most of the experiment, analyzed the data, and wrote the manuscript. Y.Y., S.X., Y.M., H.W., B.W., R.Z., L.W., X.G., F.G., and C.L. performed part of the experiments.

## Conflict of interests

The authors have no conflict of interests to declare.

## Funding

This work was supported by the 10.13039/501100014219National Science Fund for Distinguished Young Scholars of China (No. 81925015), the 10.13039/501100001809National Natural Science Foundation of China (No. 91649202), the Shandong Provincial Key Research & Development Program (China) (No. 2018YFJH0504), and the open fund of the State Key Laboratory of Reproductive Medicine (China) (SKLRM-K202201).

## References

[1] Agarwal A., Baskaran S., Parekh N. (2021). Male infertility. Lancet.

[2] Wu B., Yu X., Liu C. (2021). Essential role of CFAP53 in sperm flagellum biogenesis. Front Cell Dev Biol.

[3] Kierszenbaum A.L., Rivkin E., Tres L.L. (2011). Cytoskeletal track selection during cargo transport in spermatids is relevant to male fertility. Spermatogenesis.

[4] Vogl A.W., Grove B.D., Lew G.J. (1986). Distribution of actin in Sertoli cell ectoplasmic specializations and associated spermatids in the ground squirrel testis. Anat Rec.

[5] Ozaki-Kuroda K., Nakanishi H., Ohta H. (2002). Nectin couples cell-cell adhesion and the actin scaffold at heterotypic testicular junctions. Curr Biol.

