# Supplementary material for: CCDC178-mediated cytoskeleton assembly is required for spermiogenesis in mice
Source: Genes Dis. 2023 Sep 29;11(6):101132. doi: 10.1016/j.gendis.2023.101132 (PMC11327391; doi:10.1016/j.gendis.2023.101132)
Supplement: Multimedia component 1 [file mmc1.docx]

Supplementary Materials

**CCDC178-mediated cytoskeleton assembly is required for spermiogenesis in mice**

Fucheng Dong^1,7#^, Xiuge Wang^3#^, Tao Huang^4,5,6#^, Yingying Yin^4,5,6^, Sai Xiao^1,7^, Yanjie Ma^1,7^, Huafang Wei^1,2,7^, Bingbing Wu^1,7^, Ruidan Zhang^1,7^, Liying Wang^1,2,7^, Xuejiang Guo^8^, Fei Gao^1,7^, Chao Liu^1,2,7^, Hongbin Liu^4,5,6🖂^, Jianguo Zhao^1,7 🖂^, Wei Li^1,2,7🖂^

Correspondence to leways@gwcmc.org

**This PDF file includes:**

Supplementary Fig. S1 to S4

Materials and Methods

**
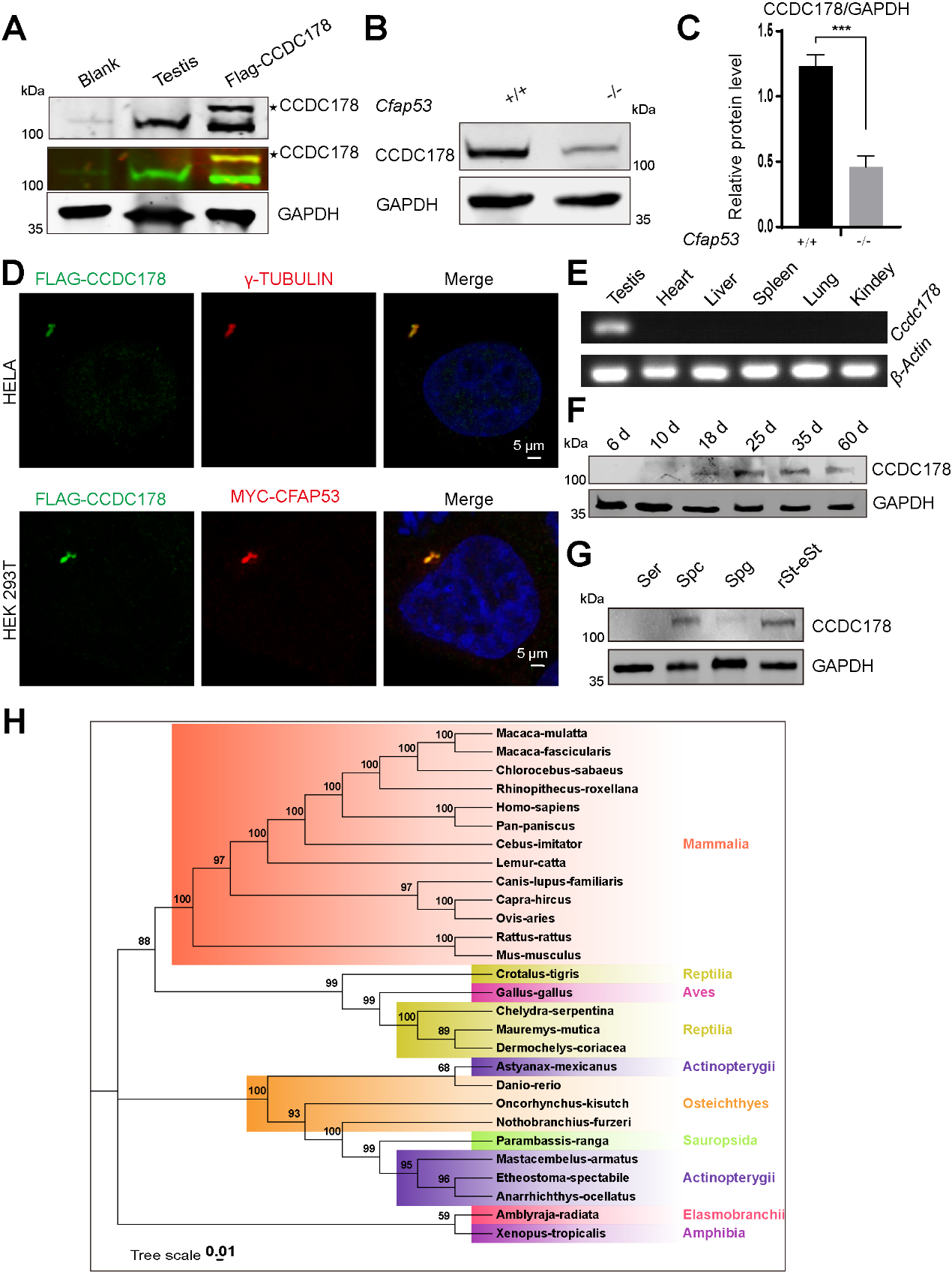
**

**Supplementary Fig. S1.**

CCDC178 is identified as a CFAP53-interacting protein that is predominantly expressed in the testes.

**(A)** Validation of the CCDC178 antibody. The CCDC178 antibody was affinity purified with immobilized antigens, and its specificity was verified by immunoblotting using extracts of HEK 293T cells transfected with pRK-Flag*-Ccdc178*. Western blotting with a commercial anti-Flag antibody. The anti-FLAG antibody recognizes a specific band of approximately 100 kDa. GAPDH served as a loading control. Asterisks indicate the right positive bands. **(B)** Immunoblot analysis of CCDC178 proteins in *Cfap53* knockout mice. GAPDH was used as the loading control. **(C)** Band intensity analysis of **(B)** n=3 independent experiments. **(D)** The localization of CCDC178 in HeLa and HEK 293T cells was analyzed by cotransfecting pRK-Flag-*Ccdc17*8 and pCS2-Myc-*Cfap53* into the cells, followed by detection with immunofluorescence staining. γ-Tubulin was used as the centrosome marker. The nucleus was stained with DAPI (blue). **(E)** The mRNA expression of *Ccdc178* in various mouse tissues. *β-actin* gene expression was used as a loading control. **(F)** Immunoblotting analysis of CCDC178 protein from various postnatal testes. GAPDH was used as a loading control. **(G)** Immunoblotting analysis of CCDC178 protein from somatic cell and different types of germ cells in the testis. GAPDH was used as a loading control. Ser, Sertoli cell, Spc, spermatocyte, Spg, spermatogonia, rSt-eSt, round spermatid and elongated spermatid. **(H)** Phylogenetic trees were constructed for different phyla such as Mammalia, Reptilia, Aves, Actinopterygii, Osteichthyes, Sauropsida, Elasmobranchii, and Amphibia. The scale bar is 0.01.

**
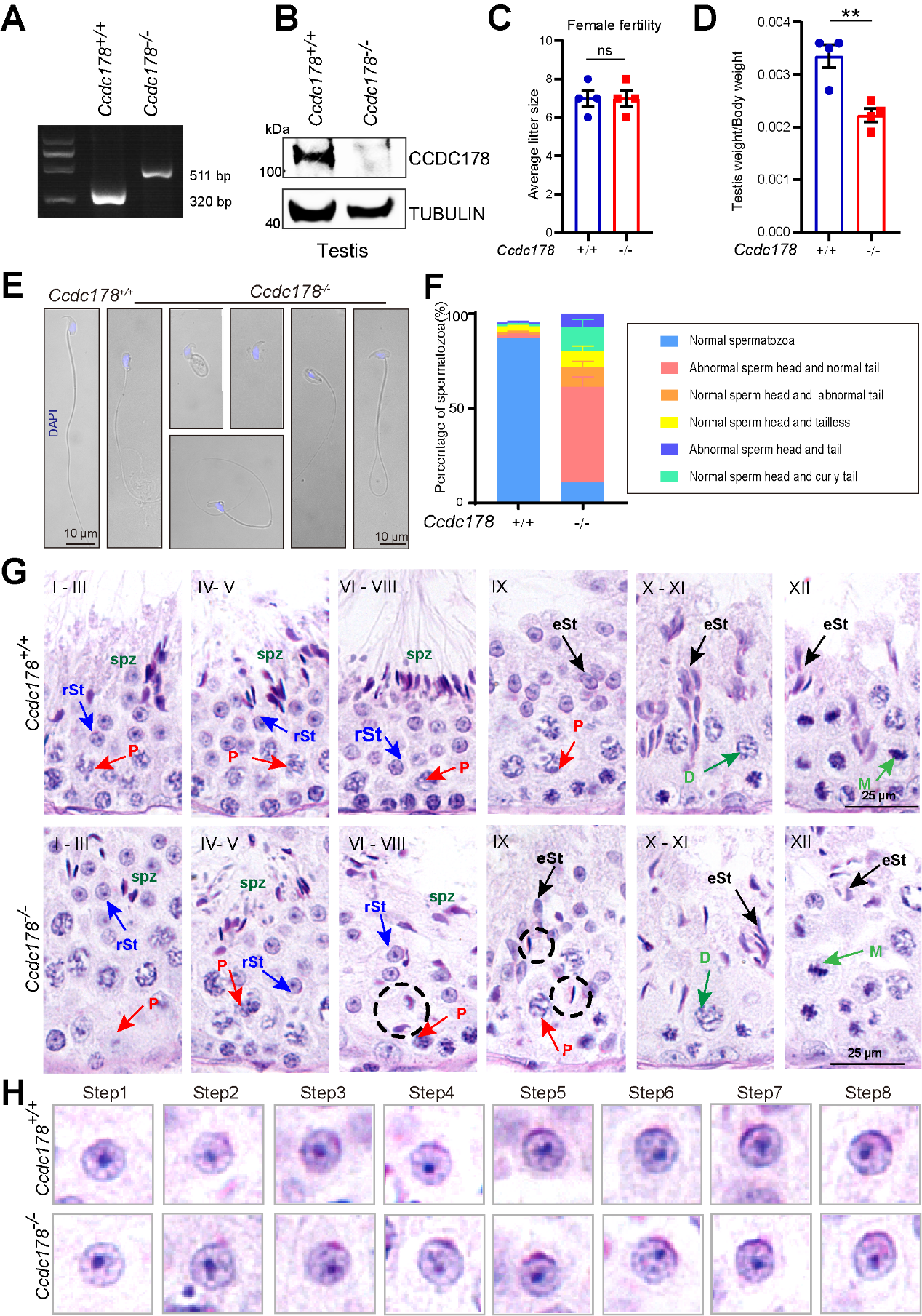
**

**Supplementary Fig. S2**.

CCDC178 is essential for male fertility.

**(A)** Genotyping of *Ccdc178* mice by PCR. **(B)** Validation of *Ccdc178* knockout in mouse testes by western blotting. Tubulin was used as a loading control. **(C)** The average litter size of *Ccdc178^+/+^* and *Ccdc178^-/-^* female mice mated with WT males. **(D)** The ratio of testis weight to body weight from the *Ccdc178^+/-^* and *Ccdc178^-/-^* mice. ns, not significant; *P* < 0.01 (**); *P* < 0.001 (***); Student’s t test with a paired two-paired distribution. **(E)** The morphology of *Ccdc178^+/+^* and *Ccdc178^-/-^* sperm was observed by staining the nucleus with DAPI. **(F)** Quantification of different categories of abnormal spermatozoa (n=3 independent experiments). Data are presented as the mean±SD. **(G)** PAS-haematoxylin staining analysis of the testis seminiferous tubule cross-sections of *Ccdc178^+/+^* and *Ccdc178^-/-^* mice. Circles show abnormal spermatids in *Ccdc178^-/-^* mice. Arrows highlight germ cells at various stages of spermatogenesis. P, pachynema; D, diplonema; M, mitotic period; eSt, elongated spermatid; rSt, round spermatid; spz, spermatozoa. **(H)** Morphology of step 1-8 spermatids in *Ccdc178^+/+^* and *Ccdc178^-/-^* testes stained with PAS-haematoxylin.

**
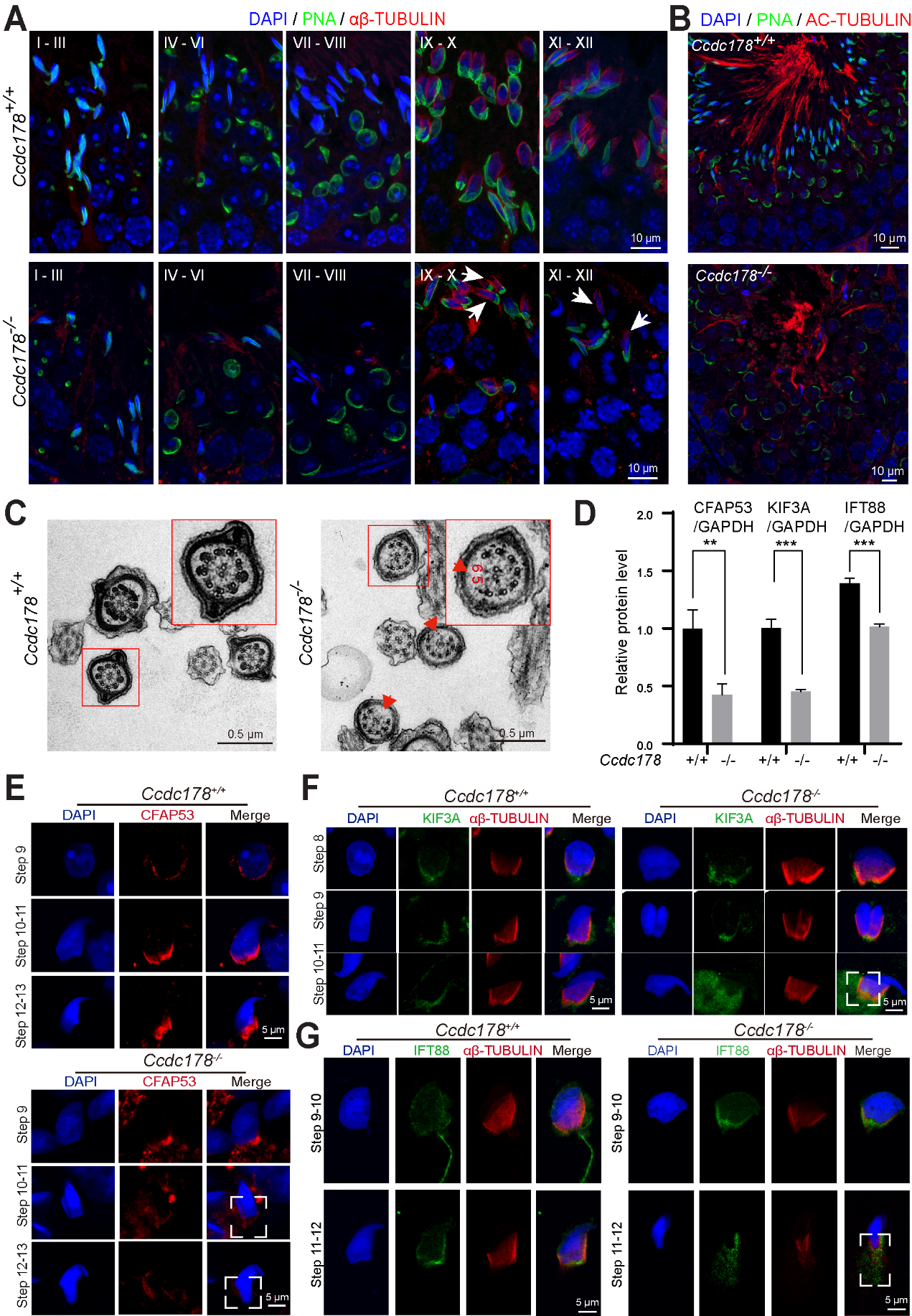
**

**Supplementary Fig. S3**.

Abnormalities in manchette and flagellum biogenesis in *Ccdc178^-/-^* mice.

**(A)** The manchette and flagellum formation abnormalities in *Ccdc178^-/-^* mice. The manchette and acrosome in *Ccdc178^+/+^* and *Ccdc178^-/-^* mouse testes spermatids stained with anti-α/β-tubulin (red) and PNA lectin histochemistry (green). The nucleus was stained with DAPI (blue). **(B)** Sperm flagellum structure at stages VI-VIII in *Ccdc178^+/+^* and *Ccdc178^-/-^* testis stained with anti-acetylated tubulin (red). The acrosome was stained with PNA lectin histochemistry (green), and the nucleus was stained with DAPI (blue). **(C)** Disruption of the “9+2” structure of sperm flagella in the *Ccdc178^-/-^* testis. TEM analyses of flagella cross sections in testes from *Ccdc178^+/-^* and *Ccdc178^-/-^* mice. Red arrows indicate the disruption of the “9+2” structure of sperm flagella. Scale bar, 500 nm. **(D)** Band intensity analysis of **Fig. 1M** (n=3 independent experiments). **(E)** Immunofluorescence staining of CFAP53 (red) in testis squash samples from *Ccdc178^+/+^* and *Ccdc178^-/-^* mice. Steps 9-12 spermatids showed abnormal CFAP53 localization in *Ccdc178^-/-^* mice (white boxes). **(F) (G)** Immunofluorescence staining of KIF3A (green) and IFT88 (green) with anti-α/β-tubulin (red) on testis squash samples from *Ccdc178^+/+^* and *Ccdc178^-/-^* mice, respectively. Step 9-12 spermatids showed abnormal KIF3A and IFT88 localization in *Ccdc178^-/-^* mice (white boxes).

**
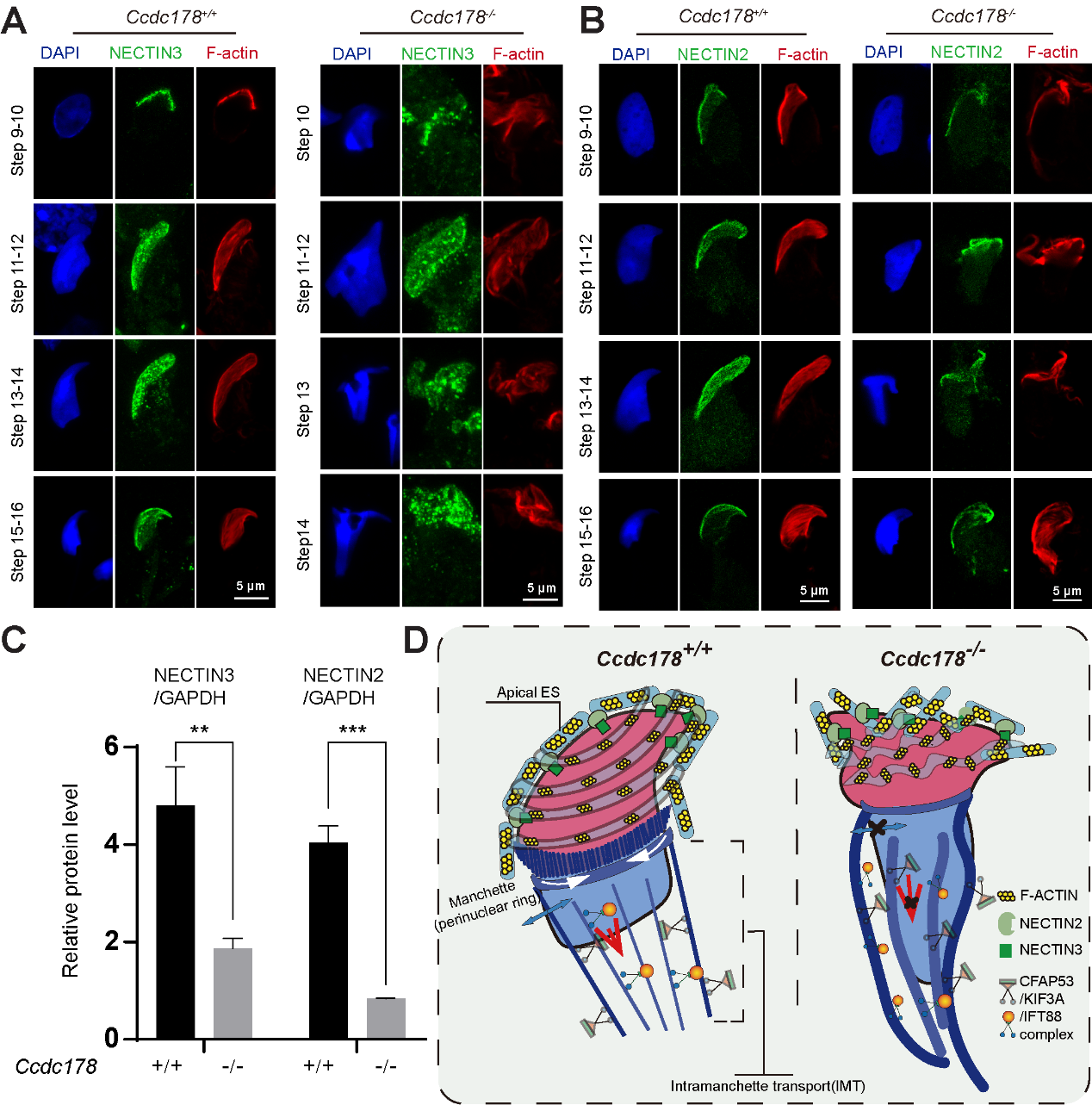
**

**Supplementary Fig. S4**.

Abnormal actin cytoskeletal structure and decreased NECTIN3/NECTIN2 in *Ccdc178^-/-^* mice.

**(A)** Immunofluorescence staining with antibodies against NECTIN3 (green) and F-actin (red) in spermatids at different developmental steps from *Ccdc178^+/^*^+^ and *Ccdc178^-/-^* adult mice. The nucleus was stained with DAPI (blue). **(B)** Immunofluorescence staining with antibodies against NECTIN2 (green) and F-actin (red) in spermatids at different developmental steps of *Ccdc178^+/+^* and *Ccdc178^-/-^* adult mice. The nucleus was stained with DAPI (blue). **(C)** Band intensity analysis of **Fig. 1S** (n=3 independent experiments). **(D)** Proposed functions of CCDC178 during spermiogenesis. CCDC178 participates in IMT by interacting directly with the CFAP53/KIF3A/IFT88 complex in the manchette, and it also participates in apical ES assembly by affecting NECTIN3/NECTIN2. The knockout of *Ccdc178* leads to abnormalities in the manchette and apical ES, resulting in defects in sperm head shaping and spermiation.

**Materials and Methods**

**Plasmids**

Mouse *Ccdc178, Cfap53*, *Ift88*, and *Kif3a* were obtained from mouse testis cDNA. These constructs were cloned into the pCS2-Myc vector or the pRK-Flag vector using the Cloning Kit (Vazyme, China), respectively. Theses vectors were obtained from BioVector NTCC.

**Animals**

All animal experiments and experimental protocols were conducted in accordance with the guidelines established by the institutional animal care and use committee (IACUC). The IACUC protocols (approved number: IOZ-IACUC-2023-181) of the Institute of Zoology, Chinese Academy of Sciences, as well as the IACUC of the Hospital for Reproductive Medicine, Shandong University, were followed.

**Generation of *Ccdc178*-knockout mice**

The *Ccdc178-*knockout mouse model was generated by CRISPR/Cas technology using the gRNA target sequence (gRNA1: GTCAGGGACGGGTCTTAATTTGG; gRNA2: ACAAGTATGGACTGCTTTCGTGG). We obtained *Ccdc178-*knockout mice through the deletion of exon 6 and exon 7 of the *Ccdc178* gene (Ensembl ID: ENSMUSG00000024306). The mice were genotyped by PCR and DNA sequencing analysis. The PCR primers used for genotyping were as follows: F1, 5’-GACTCCGTTGGTGTTCTGGTAG-3’; R1 5’-CTTCACATCTGCCCATTTC-3’; R2, 5’-CCAACAAAACATTCAGCCTAGA-3’. The wild-type allele generated a 320 bp band, the homozygotes produced a 511 bp band, and the heterozygotes produced 320 bp and 511 bp bands.

**Tissue phenotype and histological analysis**

After weighing the body, the male mice were euthanized by cervical dislocation. The tissue samples for mRNA and protein analysis were dissected and immediately frozen in liquid nitrogen. The testis and cauda epididymis were fixed in Bouin’s solution or 4% paraformaldehyde (PFA) solution overnight. Fixed tissues were dehydrated, vitrified, and embedded in paraffin. The paraffin-embedded testis and cauda epididymis were sectioned at 5 μm and detected with H&E and PAS-haematoxylin analysis according to the method described previously ^1^.

**Epididymal sperm count**

The unilateral cauda epididymis of mice was dissected and minced into small pieces in 1 ml prewarmed human tubal fluid (HTF) media (Millipore, USA) and incubated for 15 min at 37°C (5% CO_2_). The released spermatozoa were counted by calculating 3 replicates using a haemocytometer under a microscope (Primo Star, ZEISS, Germany).

**RNA extraction and RT‒PCR**

Total RNA was extracted from tissues using TRIzol reagent. cDNA was synthesized by the PrimeScript^TM^ RT Reagent Kit (TaKaRa, China,). The expression level of *Ccdc178* in the tissue was analyzed by RT‒PCR using the primers (F: 5’-AGCAGTACTGTGAAGAGAGGA-3’ and R: 5’-GCCATTTCTGGTCCAGCCTT-3’). Here, *β-actin* was used as a control gene using the primers (F: 5’-GGTGGGAATGGGTCAGAAGG-3’ and R: 5’-GTACATGGCTGGGGTGTTGA-3’). RT-PCR was performed under the following conditions: 94°C for 4 min, 25 cycles at 94°C for 30 s, 58°C for 30 s and 72°C for 20 s, and 72°C for 10 min.

**Antibodies**

The primary antibodies used for immunoblotting or immunofluorescence analysis of various proteins in this study were as follows: Rabbit anti-CCDC178 antibody (aa 304-520) was generated by Dia-an Biotech (Wuhan, China) and used at a 1:1000 dilution for western blotting. mouse anti-GAPDH (1:5000, ABclonal, AC003) and Rabbit anti-α-tubulin (1:1000, ABclonal, AC003) were used for immunofluorescence. Mouse anti-α/β-tubulin (1:200, Abcam, ab44928) and mouse anti-acetylated-tubulin (1:400, Sigma-Aldrich, T7451) were used for immunofluorescence. Mouse anti-MYC (1:1000, BE2011, EASYBIO), mouse anti-FLAG (1:1000, Abmart, M20008), mouse anti-NECTIN3(sc-271611, Santa Cruz), rabbit anti-KIF3A (Proteintech, 13930-1-AP), and rabbit anti-IFT88(Proteintech, 13967-1-AP) were used at a 1:100 dilution for immunofluorescence and at a 1:1000 dilution for western blotting. Rabbit anti-NECTIN2(ABclonal, A9622) was used at a 1:50 for immunofluorescence and at a 1:1000 dilution for western blotting. Mouse anti-CFAP53(1:20 for immunofluorescence and 1:1000 for western blotting) was produced by Dia-an Biological Technology Incorporation (Wuhan, China). The secondary antibodies were goat anti-rabbit FITC (1:200, Zhong Shan Jin Qiao, ZF-0311) and goat anti-mouse TRITC (1:200, Zhong Shan Jin Qiao, ZF-0313) for immunofluoresence. Additionally, the following reagents were used for immunofluorescence: Alexa Fluor 488 conjugate of Lectin PNA (1:400, Thermo Fisher Scientific, L21409) and TRITC conjugate of Phalloidin (1:200, Yeasen, 40734ES75).

**Western blotting**

Mouse tissues were ground and further extracted by ultrasonication in RIPA buffer (Solarbio, China) with 1% (v/v) protease inhibitor cocktail (Roche, Switzerland). After incubating the samples on ice for 30 min, the protein lysates were centrifuged at 12,000 × g for 20 min at 4°C to obtain supernatants. Protein samples of tissues were separated on SDS‒PAGE gels and transferred to nitrocellulose (NC) membranes. After blocking in 5% milk for 0.5 h, the membranes were incubated with primary and secondary antibodies. The membranes were quantitated using an ODYSSEY Imaging System (LI-COR Biosciences, USA).

**Immunofluorescence**

Frozen testis sections (7 μm) were fixed with 4% PFA at room temperature for 10 min and perforated with 0.5% (vol/vol) Triton X-100 for 10 min. After blocking sections with 5% (wt/vol) BSA, the sections were incubated with primary antibody at 4°C overnight, followed by incubation with secondary antibody for 1 h. The cell nuclei were stained with DAPI for 5 min for F-actin or PNA staining, and sections were further incubated with TRITC-conjugated phalloidin or FITC-conjugated lectin peanut agglutinin (PNA) for 1 h after secondary antibody. Images were taken with an LSM SP8 confocal microscope (Leica, Germany).

**Transmission electron microscopy (TEM)**

The adult testis tissues were treated overnight with fixation buffer containing 1.5% glutaraldehyde, 1.5% PFA and 0.1 M cacodylic acid sodium salt trihydrate. The fixed samples were cut into pieces and washed with 0.1 M cacodylic acid sodium salt trihydrate. The samples were further fixed with 1% OsO_4_ and gradually dehydrated through a concentration gradient series of acetone and Epon resin mixtures to embed in 100% resin. The ultrastructure of the samples was captured by transmission electron microscopy (JEOL, Japan).

**Immunofluorescence of testicular germ cells**

The mouse testis was dissected and fixed with a solution of 2% paraformaldehyde in 0.05% PBST (PBS with 0.05% Triton X-100) for a duration of five minutes at room temperature. Following fixation, the samples were carefully placed onto glass slides and gently flattened by adding a coverslip. Subsequently, the samples were rapidly frozen using liquid nitrogen, and the slides were stored at -80°C for subsequent immunofluorescence experiments^2^. For the preparation of the slides for immunofluorescence, the coverslips were meticulously removed, and the slides were subjected to three washes with PBS to ensure optimal cleanliness. Then, the slides were treated with 0.1% Triton X-100 for 10 min and rinsed with PBS three times again. To minimize nonspecific binding of the primary antibody, the slides were then blocked using a 5% bovine serum albumin solution for a period of one hour. Subsequently, the primary antibody was applied to the sections and allowed to incubate at 4°C.The nuclei were stained using DAPI. Immunofluorescence images were captured promptly using an LSM SP8 microscope (Leica, Germany).

**Coimmunoprecipitation (co-IP)**

The *Ccdc178,* *Cfap53*, *Ift88*, and *Kif3a* genes were cloned into the pRK (Flag tag) or pCS2 (Myc tag) plasmid. These plasmids were transiently cotransfected into human epithelial kidney 293T (HEK 293T) cells. Transfected cells were collected and lysed in TAP buffer (50 mM HEPES-KOH, 100 mM KCl, 2 mM EDTA, 10% glycerol, 0.1% NP-40 10 mM NaF, 0.25 mM Na_3_VO_4_, 50 mM glycerolphosphate, pH 7.5) plus 2 mM DTT, 0.1 mM PMSF and 1× protease inhibitor cocktail for 30 min on ice. Then, the cell lysates were centrifuged at 12,000 × g for 30 min at 4°C, and the supernatants were incubated with anti-MYC or anti-FLAG antibodies overnight at 4°C. Protein A beads were added for 2.5 h at 4°C. After washing, the beads were eluted with 1% SDS buffer for further experiments.

**Immunofluorescence of the apical ES**

The immunofluorescence analysis of testicular fragments was performed as previously described with minor modifications. The testes were fixed with 4% PFA at room temperature for 2 h and decapsulated in PBS. Seminiferous tubules were dissected using scalpels and then gently aspirated through an 18-gauge needle to fragment the seminiferous epithelium. After centrifugation at 800 × g for 5 min, the supernatant was collected and concentrated. The fragments were resuspended in a small volume of PBS and placed onto slides for 10 min. Excess fluid was removed, and the slides were air-dried. After washing with PBS three times and blocking with 5% bovine serum albumin, primary antibodies were added and incubated overnight at 4°C followed by incubation with secondary antibodies. The nuclei were stained with 4′,6-diamidino-2-phenylindole (DAPI), and images were captured immediately using an LSM SP8 microscope (Leica, Germany).

**Statistical analysis**

All statistical analyses were conducted using GraphPad Prism software (version 8.02, San Diego, USA) utilizing unpaired two-tailed Student’s t tests and one-way ANOVA. Error bars represent the standard error of the mean (SEM) with a ± sign. Statistical significance was determined when the *P* value was less than 0.05 (*), 0.01 (**) or 0.001 (***).

1. Lu LY, Wu J, Ye L, Gavrilina GB, Saunders TL, Yu X. RNF8-dependent histone modifications regulate nucleosome removal during spermatogenesis. *Dev Cell* 2010; 18(3): 371-384. doi: 10.1016/j.devcel.2010.01.010.

2. Wellard SR, Hopkins J, Jordan PW. A Seminiferous Tubule Squash Technique for the Cytological Analysis of Spermatogenesis Using the Mouse Model. *J Vis Exp* 2018;6(132):56453.  doi: 10.3791/56453.
